# The Effectiveness of Sterile Wound Drapes in the Prevention of Surgical Site Infection in Thoracic Surgery

**DOI:** 10.1155/2019/1438793

**Published:** 2019-02-11

**Authors:** Kemal Karapınar, Celalettin İbrahim Kocatürk

**Affiliations:** Department of Thoracic Surgery, Yedikule Teaching Hospital for Chest Diseases and Thoracic Surgery, Istanbul, Turkey

## Abstract

**Background:**

The rate of surgical site infections (SSIs) has decreased in parallel to advances in sterilization techniques. Such infections increase morbidity and hospitalization costs. The use of iodine-impregnated sterile wound drapes (SWDs) is recommended to prevent or reduce the incidence of these infections. However, there is a paucity of data regarding their use in thoracic surgical procedures. The aim of the present study was to evaluate the effectiveness of sterile wound drapes in the prevention of these infections and the effects on hospitalization costs.

**Methods:**

Perioperative iodine-impregnated SWDs have been used since January 2015 in the Thoracic Surgery Clinic of our hospital. A retrospective evaluation was made of patients who underwent anatomic pulmonary resection via thoracotomy with SWD in the period January 2015–2017, compared with a control group who underwent the same surgery without SWD in the 2-year period before January 2015. Factors that may have increased the risk of surgical site infection were documented and the occurrence of SSI was recorded from postoperative follow-up data. The cost analysis was performed as an important criterion to investigate the benefits of SWD.

**Results:**

Evaluation was made of 654 patients in the study group (n:380) using SWD, the operation time was significantly longer, and perioperative blood transfusion was significantly higher, whereas treatment costs (p=0.0001) and wound culture positivity (p=0.004) were significantly lower and less surgical wound debridement was performed (p=0.002).

**Conclusion:**

The findings suggest that the use of sterile wound draping in thoracic surgery procedures reduces surgical site infections and hospitalization costs.

## 1. Introduction

Surgical site infections (SSIs) have played an important role in the historical evolution of medical therapy [1]. A significant decrease has been noted in such infections since the advent of the aseptic approach in surgical interventions by Lister in the 19th century [2]. Surgical site infection is a condition that affects the skin, subcutaneous tissues, and other tissues above the fascia and is characterized by clinical evidence of an infection, purulent discharge, growth in wound culture, or the presence of inflammation findings in the first 30 days after surgery [3]. The prevalence of SSIs has decreased in parallel to the advances in sterilization techniques. However, it still occurs in 15% of clean wounds and 30% of contaminated wounds [4]. SSI is the most important health concern that increases morbidity and mortality rates after surgery, length of hospital stay, and hospitalization costs [5, 6]. Risk factors for the development of SSI include patient age, immune status, presence of malignancy, history of local or systemic infection before surgery, a history of hospitalization in the preoperative period, shaving and cleaning of the incision site before surgery, operation time, intraoperative blood replacement, and length of hospital stay after surgery [7]. The use of sterile wound drapes (SWDs) has been recommended by some to decrease or prevent SSI [8, 9]. However, there are limited data regarding the use of this material in thoracic surgical procedures. The aim of the present study was to evaluate the effectiveness of SWDs used to prevent surgical site infections and the effects on hospitalization costs.

## 2. Materials and Methods

The use of sterile wound draping in our clinic became standard practice on 01.01.2015. To evaluate the effectiveness of draping, a control group was formed of patients who underwent resection via thoracotomy (lobectomy, bilobectomy, and pneumonectomy) without sterile wound draping between 01.01.2013 and 31.12.2014. The study group comprised consecutive and similar patients who underwent procedures with the use of SWD between 01.01.2015 and 31.12.2016. Surgical site infection was defined as a condition that affects the skin, subcutaneous tissues, and other tissues above the fascia and is characterized by clinical evidence of an infection, purulent discharge, growth in wound culture, or the presence of inflammation findings from 3 days to 30 days after surgery. Patients were excluded from the study if they had factors that independently increased surgical site infections (chronic local or systemic infection requiring the use of steroids, previous history of hospitalization before surgery, uncontrolled diabetes mellitus manifested by a high level of glycosylated hemoglobin, and immunodeficient patients), if they were obese (Body Mass Index >30), if data were not available, if they did not wish to be included, if they died before postoperative day 30 from nonsurgical disease such as cardiac death, if they were receiving adjuvant therapy (chemotherapy or radiotherapy), or if lobectomy was applied using Video Assisted Thoracoscopic Surgery (VATS) via a mini-incision.

The patients in both groups were stratified according to the presence of risk factors and univariate analyses were performed. The effects of the development of SSIs and the use of SWDs on the hospitalization costs were investigated. Hospital costs were defined as the data reported to the social security institution, which is automatically calculated after the patient is discharged. These data include all medicines, materials, and personnel expenses used throughout hospitalization period. A record was made of patient age, gender, comorbidities, previous hospitalizations, length of stay in the surgical intensive care unit, operation time, first C-reactive protein (CRP) value in the postoperative period, cost, length of hospital stay, and the amount of blood transfused due to intraoperative hemorrhage SSI and VAC therapy. The distribution of positive results in the samples sent to the microbiology laboratory (blood, sputum, wound, and debridement culture), probability of occurrence, and operation types across the groups were evaluated to investigate the reasons for SSI in detail.

Approval for this retrospective study was granted by the Local Ethics Committee (2016/42).

### 2.1. Surgical Procedure

**Figure 1:**
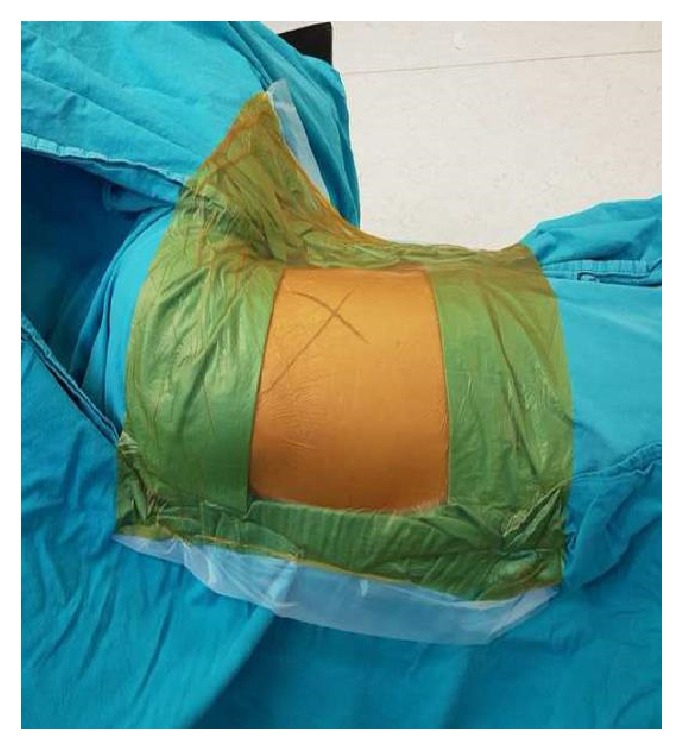
Draped surgical site with iodine-impregnated drape.

Patients who were planned to undergo surgery in our clinic were advised to have a bath one day before surgery, and the surgical site (ipsilateral hemithorax) was shaved by a ward nurse with an electric shaver just before surgery. The surgical site was cleaned with the application of iodine solution three times before starting surgery. The sterile covering was applied. A sterile gauze compress was used to remove the excess of partially dried iodine solution to allow adhesion of the barrier in the SWD group (study group). Sterile wound drapes (Ioban(®) 2, 3M Science, Minneapolis, USA) were tightly placed so as to cover all areas left uncovered by other sterile coverings (Figure 1). No additional procedure was performed to dry the iodine solution in the undraped patients (control group). The skin was closed with a stapler at the end of the procedure. All skin covers were removed in both groups, and the surgical site was cleaned with iodine solution and dressed with sterile materials. The dressings were removed after 48 hours in the absence of visible contamination, and the wound was examined. When the wound was clean, it was cleaned with iodine solution and left open. The daily physical examination was performed with inspection and palpation. No further wound dressing was performed afterwards, and the patient was advised to have a bath at four and six days. Sutures were removed on day seven. Hemogram, CRP levels, and chest X-ray were obtained in the postoperative period every other day until discharge. Infection parameters were monitored. Patients were monitored for signs of intrathoracic infection. Surgical site infection was diagnosed and treated by surgeons who were not involved in the study.

The patients were advised to come for weekly control examinations for one month after discharge (more frequently if required). Swab culture was obtained from the incision site in every patient showing signs of surgical site infection in the postoperative period. In the presence of accompanying leukocytosis, elevated CRP levels, and fever, antibiotics were initiated after consultation with an infectious disease specialist without waiting for the culture results. When the signs that were reported above were not observed, local wound infection was treated with known surgical techniques. Antibiotherapy was modified according to the results of the susceptibility test if the growth in the culture was not judged as contamination. Open wound dressing, debridement, and Vacuum Assisted Closure (VAC) were used to treat local surgical wound infections [10]. In debridement, the wound is thoroughly cleaned with the removal of all hyperkeratotic (thickened skin or callus), infected, and nonviable (necrotic or dead) tissue, foreign debris, and residual material from dressings.

### 2.2. Statistical Analysis

Data obtained in the present study were analysed statistically using NCSS (Number Cruncher Statistical System) 2007 Statistical Software (Utah, USA) package. Together with descriptive statistics (mean, standard deviation), distribution of the data was tested with the Shapiro-Wilk normality test. The Independent t-test was used in the paired comparisons of normally distributed data, and the Mann-Whitney U-test was used for the paired comparisons of nonnormally distributed data. The Chi-square test was used to compare qualitative data. A value of p<0.05 was accepted as statistically significant.

## 3. Results

Within the specified period, 685 patients were identified for inclusion in the study. A total of 31 patients were excluded: 8 patients with factors increasing SSIs, 6 patients with unavailable data, 12 patients that dropped out of the study, and 5 patients that died before day 30. Therefore, evaluation was made of 654 patients, as 274 in the control group and 380 in the study group. It was observed that patients with malignancy were predominant in both groups.

There was no statistically significant difference in respect of mean age, gender, morbidities, or previous hospitalizations between the control and study groups (p=0.06, p=0.784, p=0.557, and p=0.144) (Table 1). There was no statistically significant difference in the mean length of stay in the intensive care unit (day) between the control and study groups (p=0.590). The mean operation time was significantly shorter in the control group than in the study group (p=0.0001). No significant difference was determined in respect of the mean CRP level between the control and study groups (p=0.617). The mean hospitalization cost was significantly higher in the control group than in the study group (p=0.0001). There was no statistically significant difference in the mean length of hospital stay between the groups (p=0.093). The mean blood transfusion use during surgery was significantly lower in the control group than in the study group (p=0.0001). The rate of surgical site infections was significantly higher in the control group than in the study group (p=0.001). The use of Vacuum Assisted Closure was higher in the control group but not at a statistically significant level (p=0.01). There was no statistically significant difference between the groups in respect of the mean blood culture positivity (p=0.311). The likelihood of blood culture positivity was 1.41-fold (0.72-2.77) higher in the control group than in the study group. Sputum culture positivity was 1.15-fold (0.55-32.37) higher in the control group than in the study group, but the difference was not statistically significant (p=0.706). Wound culture positivity was significantly higher in the control group than in the study group (p=0.004). The likelihood of wound culture positivity was 3.27-fold (0.40-7.63) higher in the control group than in the study group. The rates of wound debridement were significantly higher in the control group than in the study group (p=0.002). The likelihood of wound debridement culture positivity was 7.91-fold (1.74-35.97) higher in the control group than in the study group (Table 2). There was a statistically significant difference in the distribution of operation types between the control and study groups (p=0.0001) (Table 3). The rates of bronchial sleeve lobectomy, bronchovascular sleeve lobectomy, and lung resection after neoadjuvant chemoradiotherapy were lower, and the rate of extended lung resection using an intrapericardial approach was higher in the control group than in the study group.

## 4. Discussion

Recent opinions have stated that the use of SWDs in patients who undergo surgery reduces the incidence of postoperative SSIs as well as the hospitalization costs [9]. There are, however, also studies that suggest the contrary. Some studies have reported no difference between wound site sterilization using classic methods and disinfection with SWDs. SWDs are not routinely used in the practice of thoracic surgery. Thus, to the best of our knowledge, there has not been any research in literature that has included a large number of patients. Since January 2015, the use of SWDs has become routine practice in our clinic based on reports in literature supporting their use [8, 9, 11, 12]. The present study was planned in our clinic, which mostly performs surgery for non-small cell lung cancer. Prospectively archived records during the treatment of patients were retrospectively reviewed. Despite decreasing rates, lung cancer is still more commonly seen in males [13]. No significant difference was observed between the current study groups in respect of age, gender, morbidity, or previous hospitalization (p=0.06, p=0.78, p=0.55, and p=0.14, respectively) (Table 1). The fact that there is no difference in these factors demonstrates no difference in preoperative risk factors for SSI between the groups. However, the results of the study showed a significantly higher rate of SSI in the control group (p<0.001). The probability of developing decubitus ulcer as a result of pressure and Ventilator Associated Pneumonia (VAP) is known to increase with prolonged length of stay in the intensive care unit [14]. Decubitus ulcer may exhibit symptoms similar to SSI, and both could have affected the reliability of the study due to wound site culture positivity and the requirement for debridement. VAP is also important as it may cause an increase in CRP levels and blood culture positivity as does SSI. In the current study, there was no significant difference between the groups in respect of the length of stay in the intensive care unit (p=0.59). This lack of difference increased the reliability of this study. Extended operation time is a factor that may have affected sterility due to distraction in the surgical team. It can therefore be regarded as one of the causes that increase SSIs. The operation time was significantly longer in the study group (p<0.0001), which can be attributed to a significantly higher rate of long-awaited surgeries (bronchial sleeve lobectomy, bronchovascular sleeve lobectomy, and lung resections after neoadjuvant chemoradiotherapy) in the study group (Table 2). The fact that wound culture and debridement culture positivity rates were lower in the same study group can be seen as the success of the surgical team. A high CRP level is a general indication of an infection in the body. CRP levels may increase false positive results, as CRP levels are elevated in SSIs. The lack of significant difference between the two groups in respect of this parameter also increased the reliability of the study (p=0.617). Decreased hospitalization costs is an important parameter to evaluate the effectiveness of treatment. Bejko et al. reported that an iodine-impregnated drape (Ioban(®) 2 drape) decreases both SSIs and hospitalization costs in patients undergoing cardiac surgery when compared to iodine-free drapes [9]. In the current study, that significantly lower hospitalization costs were determined with the use of iodine-impregnated drape is a criterion indicating the effectiveness of the treatment (p<0.0001). Another reason for this could be the shorter length of hospital stay in the study group, although the difference was not statistically significant (p=0.093). Blood transfusion during surgery may increase the risk of SSI [4]. As previously stated and depicted in Table 2, procedures with a prolonged operation time were more commonly performed in this study group. Increased operation time and difficulty level may increase the risk of hemorrhage. Blood transfusion during and after surgery is directly proportionate to the presence or extent of hemorrhage. In the current study, a significantly higher number of blood transfusions were performed in the study group. However, that there was no difference in infection parameters, including blood culture positivity (p=0.311) and CRP levels, suggests that events such as transfusion reaction, which may be misdiagnosed as infection, were at similar levels. These similarities also increased the reliability of the study. Positive sputum culture is a finding of pneumonia, which is a known cause of morbidity in patients who undergo thoracic surgical procedures following neoadjuvant therapy [15]. A positive sputum culture can therefore affect many other parameters, including the length of hospital stay, length of stay in the intensive care unit, and CRP level. There was no significant difference between the groups in the current study in respect of sputum culture positivity. From the results of this study, after excluding other infection parameters, it can be suggested that the parameters affecting SSI are wound site culture positivity and wound debridement. Both of these parameters were significantly lower in the study group where iodine-impregnated SWD was used (p<0.004 and p<0.002).

The first study to use SWD was performed by Lewis et al. [12]. In that study, iodine-impregnated SWD, iodine-free SWD, and only povidone-iodine application were compared in patients who underwent inguinal hernia surgery, and iodine-impregnated SWD was found to be more effective. In a review of seven studies by Webster et al. [4], the findings showed that SWDs did not affect hospitalization costs and infection rates, whereas iodine-free dressings would increase SSIs. In a manuscript published 8 years later as a continuation of the Webster review, no supplemental data were reported due to lack of new randomized and controlled studies [16]. The reason for this could be that surgeons refrain from using a method which has previously yielded poor results. The same study also reported an increased length of hospital stay. In the present study, there was no change in the length of hospital stay, but there was a decrease in hospitalization costs. It was concluded from the current study results that SWD is effective. In the study by Casey et al. [8], the use of iodine-impregnated SWDs, when compared to other methods, was associated with lower rates of Methicillin Resistant* Staphylococcus aureus* (MRSA). The present study defined wound site infection with culture positivity of the pathogenic bacteria, the presence of purulent discharge, and debridement, and no agent-specific approach was used. As a continuation of this study, the authors may further investigate a specific agent, such as MRSA. In the study by Casey et al. SWDs were found to be ineffective after procedures such as appendectomy, which are potentially prone to infections. In the current study, although the potential risk of infection was higher because of the longer operation time and higher blood transfusion rate, the rate of SSIs was lower with the use of iodine-impregnated SWD. Bejko et al. [9] reported a lower rate of SSI and lower hospitalization costs with the use of SWD in patients who underwent cardiac surgery, which encouraged the current study authors to replicate that study on patients who underwent thoracic surgery. The results of this retrospective study showed that when other sources of infection were excluded, SSIs characterized by wound culture positivity and wound debridement occurred at a significantly lower rate with the use of SWDs (p<0.004 and p<0.002). Similarly, hospitalization costs were also lower (p<0.0001). Thus, the use of SWDs can be recommended to decrease SSIs. Although the length of stay in hospital was the same, the cost was reduced, because the number of SSIs was lower, with an associated reduction in expensive treatments such as debridement and VAC. In a study by Milandt et al. [17], it was reported that bacterial recolonization did not increase in patients who underwent bilateral knee arthroscopy. Bilateral surgery is a factor that increases the operation time.

The limitations of this study were that randomization could not be performed because the study was retrospective, and the control and study groups were operated on consecutively but at different times. Although it can be viewed as a limitation that patients with malignant or benign diseases were mixed in both groups, the type of operation was the same and the exclusion of adjuvant therapy patients (chemo- or radiotherapy) ensured homogeneity of the groups.

That the probability of infection did not increase with increasing operation time demonstrates the reliability of the present study. The use of SWDs can be recommended as effective in the reduction of SSIs in lengthy thoracic surgical procedures, most of which are performed by a single surgical team for oncological reasons in our clinic. The present study can be considered of value in leading the drive for future studies, given that, to the best of our knowledge, there has been no similar study in the field of thoracic surgery. To confirm the results and continue the present research, further studies are being planned in collaboration with the Departments of Microbiology and Infectious Diseases to identify the agents of infection.

## Figures and Tables

**Table 1:** Demographic characteristics and previous illness of patients.

	Control Group n:274		Study Group n:380		p
Age	60.03±11.72		58.24±12.14		0.06*∗*
Gender					
Male	219	79.93%	307	80.79%	0.784^+^
Female	55	20.07%	73	19.21%	
Morbidity (DM, HT, etc.)	80	29.20%	103	27.11%	0.557^+^
Previous hospitalization	8	2.92%	20	5.26%	0.144^+^

**Table 2:** Demographic characteristics of the participating patients.

	Control Group n:274		Study Group n:380		p	OR 95% CI
Length of Stay in the Intensive Care Unit (days)	1.33±1.25		1.48±2.48		0.590*ǂ*	
Operation Time (mins)	222.08±68.95		268.94±92.17		*0.0001 * **∗**	1.01 (1.00-1.03)
CRP	18.56±12.72		19.3±13.55		0.617**∗**	
Cost	5942±2740		4813±1996		*0.0001ǂ*	0.83 (0.78-0.98)
Length of Hospital Stay (days)	8.69±4.65		8.21±5.12		0.093*ǂ*	
Blood Usage	1.08±2.03		2.53±2.31		*0.0001ǂ*	1.56 (1.39-1.75)
SSI	25	9.12%	11	2.90%	*0.001* ^*+*^	0.30 (0.14-0.61)
VAC	10	3.65%	3	0.79%	*0.010* ^*+*^	0.21 (0.06-0.77)
Blood Culture						
Absent	256	93.43%	362	95.26%	0.311^+^	1.41(0.72-2.77)
Present	18	6.57%	18	4.74%		
Sputum Culture						
Absent	260	94.89%	363	95.53%	0.706^+^	1.15 (0.55-32.37)
Present	14	5.11%	17	4.47%		
Wound Culture						
Absent	256	93.43%	372	97.89%	*0.004* ^*+*^	3.27 (0.40-7.63)
Present	18	6.57%	8	2.11%		
Wound Debridement Culture						
Absent	263	95.99%	378	99.47%	*0.002* ^*+*^	7.91(1.74-35.97)
Present	11	4.01%	2	0.53%		

**∗**Independent samples t-test, *ǂ*: Mann-Whitney U test + Chi-square test, SSI: Surgical Site Infections, and VAC: Vacuum Assisted Closure.

**Table 3:** Distribution of the operation types in the control and study groups.

	Control Group n:274		Study Group n:380		p
Operation					
Lung resection and mediastinal lymph node resection	188	68.61%	265	69.74%	*0.0001*
Bronchial sleeve lobectomy	2	0.73%	17	4.47%	
Bronchovascular sleeve lobectomy	1	0.36%	8	2.11%	
Extended lung resections	75	27.37%	68	17.89%	
Lung resections after neoadjuvant chemoradiotherapy	8	2.92%	22	5.79%	

## Data Availability

Original data is available on request.
